# Letter from the Editor in Chief

**DOI:** 10.19102/icrm.2026.17085

**Published:** 2026-08-15

**Authors:** Devi Nair


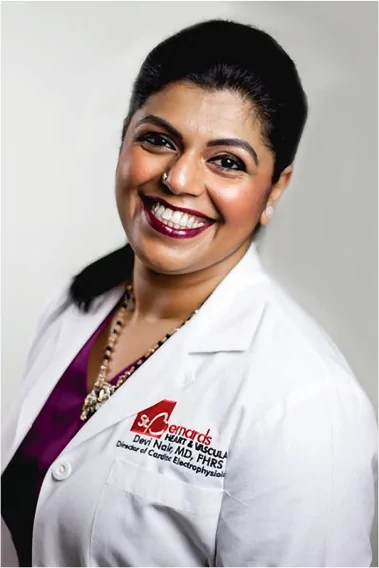


Dear Colleagues,

Welcome to the August 2026 issue of *The Journal of Innovations in Cardiac Rhythm Management*.

The end of summer is a quiet milestone for our profession. A new class of trainees is now a few months into fellowship, past the first disorientation and beginning to find their footing at the mapping system and the bedside. This year’s graduates are a few months into first jobs, learning that independence is exhilarating and lonelier than fellowship prepared them for. And, for many, the autumn certification examinations now loom on the calendar, that last formal hurdle before a hard-won credential. To all of you in these first uncertain months, a word of encouragement: the competence you are reaching for is not a destination you arrive at and keep, but a practice you renew every day, and the discomfort you feel now is simply the feeling of learning at full speed. Lean on your mentors, prepare deliberately, and be patient with yourselves.

August brought our community to Overland Park for the Kansas City Heart Rhythm Symposium (KCHRS), where I had the privilege of serving on faculty. Now in its long and distinguished run, KCHRS has become one of the most engaging gatherings on the electrophysiology calendar, precisely because it refuses the passive format. Its two days are built around debate; live and recorded cases; and the kind of frank, unscripted exchange that only happens when experienced operators are willing to disagree in public and reason their way toward common ground. The meeting opened, fittingly, with a Fellows Bootcamp and Allied Healthcare Program, and there is something clarifying about a symposium that begins by teaching its youngest attendees before turning to its most contested questions. This year’s awards captured the arc of our field with unusual clarity. William Stevenson received the Pioneer in Electrophysiology award, a fitting recognition of a body of work on re-entry, entrainment, and substrate that has shaped the very framework within which most of us still reason about ventricular tachycardia; generations of electrophysiologists are, in a real sense, practicing inside a structure he built. Steven Mickelsen received the Trailblazer award for his part in carrying pulsed field ablation from a contrarian idea to the central conversation in atrial fibrillation ablation in under a decade, an achievement that began with his refusal to accept that thermal energy was the only option. To honor in a single room the physician who taught us to understand the circuit and the one who changed how we treat it was to watch the past and the future of electrophysiology in conversation. I came away, as I always do from Kansas City, reminded that our field is at its best when it argues generously and teaches as it goes.

If KCHRS looks at where our practice stands today, the meeting I am most looking forward to this autumn asks where it is going. HRX Live 2026 convenes next month, September 18 through 20, in Atlanta, and it is unlike our other gatherings by design. Rather than the incremental refinement of established technique, HRX is devoted to the disruptive edge of our field; artificial intelligence diagnostics, digital health and wearables, next-generation mapping, and the novel energy sources reshaping ablation; and to the uncomfortable but necessary conversations between clinicians, engineers, entrepreneurs, and regulators that innovation demands. I would encourage anyone who wishes to understand the shape of electrophysiology a decade from now to attend. The manuscripts in this very issue already show that future arriving, from powered lead-extraction tools to the mapping of complex ventricular circuits.

## In This Issue

The work collected this month spans one original research study and five instructive cases and tracings, together covering lead extraction, the complications and the anatomy of ablation, electroanatomic mapping, and the fine reading of the intracardiac electrogram.

In an original research contribution, Reddy, Saggu, and colleagues^1^ from AIG Hospitals, Hyderabad, compare cut and coagulation modes of electrosurgical energy for pacemaker lead-tip extraction in a porcine model and then in a patient. The coagulation, or fulguration, setting allowed controlled dissection with minimal tissue penetration and no collateral injury and enabled complete removal of an 8-year dwell lead without complication, a promising adjunct where powered tools near the lead tip are otherwise contraindicated.

Munshi, Ahmad, Dajani, and colleagues^2^ from Saint Joseph University Medical Center report persistent right phrenic nerve palsy following pulsed field ablation with the PulseSelect™ system (Medtronic, Minneapolis, MN, USA), used for pulmonary vein and posterior wall isolation in a woman with paroxysmal atrial fibrillation, with hemidiaphragmatic elevation still present at 3 months. The relative tissue selectivity of pulsed field ablation is not the same as immunity from collateral injury, and complications this rare underscore the importance of capturing adverse events in large, prospective registries, where signals too infrequent to surface at any single center can be recognized, quantified, and acted upon.

Marins, Essebag, and Bernier^3^ from the McGill University Health Centre describe the ablation of a para-Hisian accessory pathway from the non-coronary cusp in a young man with pre-excited atrial fibrillation, after right-sided radiofrequency delivery proved unsafe near the conduction system. A retrograde aortic approach eliminated pre-excitation within seconds—a clean illustration of the non-coronary cusp as an alternative when right-sided ablation is high-risk.

Ozeke, Aras, and Topaloglu^4^ from Ankara Bilkent City Hospital use a case of sustained monomorphic ventricular tachycardia to distinguish the critical isthmus from a noncritical bystander, showing how mid-diastolic potentials and morphologically distinct QRS complexes betray double-entrance and double-exit circuit configurations. It is a compact lesson in reading the electrogram for what it says about the circuit.

Singh, Venkadesan, and colleagues^5^ from Dr. Ram Manohar Lohia Hospital, New Delhi, use a focal atrial tachycardia in a young man to press a subtle but consequential mapping point: the site of earliest activation on the electroanatomic map is not always the true focal source, and a breakthrough from a broad, deep focus can masquerade as the target. It is a cautionary reminder not to let the color map stand in for electrophysiologic reasoning.

Finally, in this month’s Tracing of the Month, Mondal, Nanda, and Muslim^6^ from The Mission Hospital, Durgapur, present an exceedingly rare irregularly irregular narrow complex tachycardia: an atrioventricular nodal re-entrant tachycardia sustained through a lower common pathway exhibiting Wenckebach periodicity. The tracing rewards the reader willing to work out why a re-entrant rhythm should appear so disorganized at the surface.

What connects the debates of Kansas City, the innovations gathering in Atlanta, and the manuscripts in these pages is a single habit of mind: the willingness to examine our tools and our assumptions honestly and to teach what we learn. I will close, as ever, with an invitation. If you have a study, a case, or a tracing that taught you something worth sharing, this journal exists to publish it, and we are always glad to see the work of trainees and of colleagues from every corner of our global community.

I am grateful, as always, to our authors, reviewers, and editorial team for their work, and to you, our readers, for your continued engagement with the journal.

Warm regards,


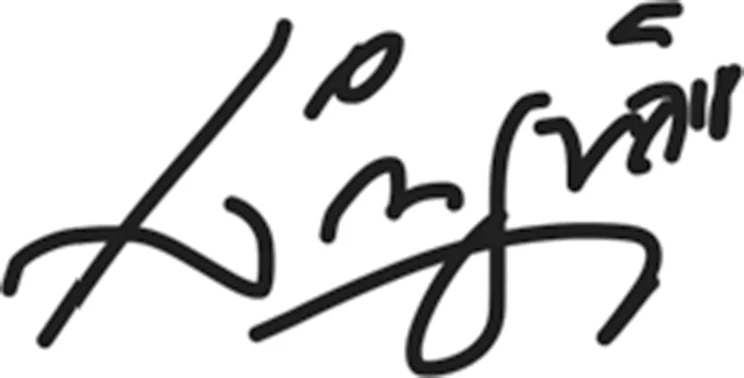


Dr. Devi Nair, md, facc, fhrs

Editor-in-Chief

*The Journal of Innovations in Cardiac Rhythm Management*

Director of the Cardiac Electrophysiology & Research,

St. Bernard’s Heart & Vascular Center, Jonesboro, AR, USA

White River Medical Center, Batesville, AR, USA

President/CEO, Arrhythmia Research Group

Clinical Adjunct Professor, University of Arkansas for Medical Sciences

Governor, Arkansas Chapter of the American College of Cardiology

drdgnair@gmail.com
